# A detailed analysis of next generation sequencing reads of microRNA expression in Barrett’s Esophagus: absolute versus relative quantification

**DOI:** 10.1186/1756-0500-7-212

**Published:** 2014-04-04

**Authors:** In-Hee Lee, Xiaoman Hong, Sharad C Mathur, Mukut Sharma, Amit Rastogi, Prateek Sharma, Lane K Christenson, Ajay Bansal

**Affiliations:** 1 Bioinformatics Core Facility, University of Kansas, Lawrence, KS, USA; 2 Department of Molecular and Integrative Physiology, University of Kansas Medical Center, Kansas City, KS, USA; 3 Department of Pathology, Veterans Affairs Medical Center, Kansas City, KS, USA; 4 University of Kansas Medical Center, Kansas City, KS, USA; 5 Veterans Affairs Medical Center, Kansas City, KS, USA; 6 The Kidney Institute, University of Kansas Medical Center, Kansas City, MO, USA; 7 Division of Gastroenterology and Hepatology, Veterans Affairs Medical Center, Kansas City, KS, USA; 8 Kansas Cancer Institute, University of Kansas Medical Center, Kansas City, KS, USA; 9 Department of Gastroenterology, University of Kansas School of Medicine, 3901 Rainbow Blvd, Kansas City, KS 66160, USA

**Keywords:** Next generation sequencing, MicroRNA, qRT-PCR, Correlation, Barrett’s esophagus

## Abstract

**Background:**

Next generation sequencing (NGS) is a state of the art technology for microRNA (miRNA) analysis. The quantitative interpretation of the primary output of NGS i.e. the read counts for a miRNA sequence that can vary by several orders of magnitude (1 to 10^7)^ remains incompletely understood.

**Findings:**

NGS (SOLiD 3 technology) was performed on biopsies from 6 Barrett’s esophagus (BE) and 5 Gastroesophageal Reflux Disease (GERD) patients. Read sequences were aligned to miRBase 18.0. Differential expression analysis was adjusted for false discovery rate of 5%. Quantitative real-time polymerase chain reaction (qRT-PCR) was performed for 36 miRNA in a validation cohort of 47 patients (27 BE and 20 GERD). Correlation coefficients, accuracy, precision and recall of NGS compared to qRT-PCR were calculated. Increase in NGS reads was associated with progressively lower Cq values, p < 0.05. Although absolute quantification between NGS reads and Cq values correlated modestly: -0.38, p = 0.01 for BE and -0.32, p = 0.05 for GERD, relative quantification (fold changes) of miRNA expression between BE &GERD by NGS correlated highly with qRT-PCR 0.86, p = 2.45E-11. Fold change correlations were unaffected when different thresholds of NGS read counts were compared (>1000 vs. <1000, >500 vs. <500 and >100 vs. <100). The accuracy, precision and recall of NGS to label a miRNA as differentially expressed were 0.71, 0.88 and 0.74 respectively.

**Conclusion:**

Absolute NGS reads correlated modestly with qRT-PCR but fold changes correlated highly. NGS is robust at relative but not absolute quantification of miRNA levels and accurate for high-throughput identification of differentially expressed miRNA.

## Findings

Next generation sequencing (NGS) is a significant advancement over hybridization-based microarrays for microRNA (miRNA) discovery. NGS can measure miRNA expression across several orders of magnitude from 1 to 10^7^. However, the quantitative interpretation of the primary output of NGS i.e. read counts for a miRNA sequence remains unclear. The current practice is to validate NGS findings by qRT-PCR [1-4]. However, the published studies have several limitations—a small number of biological samples [1,2], primarily qualitative analysis [3], introduction of bias by selection for validation of only differentially expressed miRNA by NGS [3] and lack of guidance on low-versus high-abundance transcripts [1-4]. Specifically, several unanswered questions remain. How do NGS read counts correlate with Cq values on quantitative real-time polymerase chain reaction (qRT-PCR)? Is there a threshold copy number below which miRNA detection becomes unreliable? What is the overall sensitivity and specificity of NGS for identifying the miRNA of interest? How does NGS perform at absolute quantification of a transcript expression versus relative quantification between experimental and control groups? Does detection of differential expression of miRNA in a disease state depend on transcript abundance? Barrett’s esophagus (BE) is a pre-malignant condition for rapidly increasing esophageal adenocarcinoma and is a complication of Gastroesophageal Reflux Disease (GERD) [5]. Here we present the systematic comparison of miRNA expression by NGS and qRT-PCR in well-characterized patients with BE and GERD.

## Methods

### Study design and patient selection

We previously sequenced the miRNA transcriptome in GERD and BE [6] and evaluated 14 differentially expressed miRNAs by qRT-PCR. For the current analysis, we analyzed an additional 22 miRNAs that were not differentially expressed by NGS. These additional miRNAs were randomly selected to represent the varying level of expression by NGS in GERD and BE tissues and to allow us to calculate NGS performance in an unbiased manner. Thus, we evaluated a total of 36 miRNA by qRT-PCR (Table 1). Patients with GERD and BE were selected from a prospective tissue and serum repository (Clinical Trials.gov # NCT00574327). The details of the repository, definitions and inclusion and exclusion criteria have been described previously [6]. The repository was created with approval by the Human Subjects Committee and the Research and Development Committee of the Institutional Review Board, Veterans Affairs Medical Center, Kansas City, Missouri. The repository has been annually approved since 2005. All patients sign an IRB approved informed consent prior to inclusion in the registry that allows us to store samples for future research related to GERD and BE. The approval number for the patient registry is ePROMISE PS0035 as determined under the institutional regulations. Briefly, BE is defined as presence of columnar lined esophagus on endoscopy with demonstration of intestinal metaplasia in biopsies. GERD is defined on the basis of presence of heartburn and/or regurgitation on a standardized and validated questionnaire. GERD patients are further sub-classified into those with erosive esophagitis (EE) and those without (Non-erosive reflux disease, NERD) based on the findings of esophagitis (or lack thereof) on endoscopy. To study a homogeneous population, for this study we included only those GERD patients who had EE. The initial NGS cohort was comprised of 11 patients, five with GERD and six with BE, all patients also underwent qRT-pCR. We also tested all of the 36 miRNAs in an independent cohort of 20 GERD and 27 BE patients by qRT-PCR.

**Table 1:** List of miRNA analyzed with their expression values by NGS

**miRNA**	***Average NGS read counts***	
	**GERD**	**BE**
*hsa-mir-944*	28.9	0.1
*hsa-mir-466*	20.1	4.3
*hsa-mir-365a-5p*	23.2	4.5
*hsa-mir-3065-5p*	36.3	8.3
*hsa-mir-133a*	1.9	14.4
*hsa-mir-376a-3p*	18.2	22.3
*hsa-mir-296-5p*	99.7	19.6
*hsa-mir-299-5p*	10.2	36.5
*hsa-mir-1260b*	448.5	43.4
*hsa-mir-337-5p*	7.7	71.5
*hsa-mir-542-5p*	10.7	77.6
*hsa-mir-708-5p*	967.7	78.8
*hsa-mir-196b-5p*	8.4	98.2
*hsa-mir-487b*	36.6	106.2
*hsa-mir-486-5p*	110.1	140.7
*hsa-mir-224-5p*	2052.5	210
*hsa-mir-188-5p*	210.8	288.3
*hsa-mir-338-5p*	31.4	489.5
*hsa-mir-149-5p*	3860.1	558
*hsa-mir-196a-5p*	37.7	586.6
*hsa-mir-182-5p*	1149.3	1238
*hsa-mir-378c*	1040.2	1723.2
*hsa-mir-424-5p*	491.7	1807.4
*hsa-mir-339-5p*	1430.4	2030.9
*hsa-mir-203*	90723.5	3569.2
*hsa-let-7d-5p*	3153.1	3594.9
*hsa-mir-199b-5p*	810.7	3880.1
*hsa-mir-195-5p*	1342.0	4248.0
*hsa-mir-15b-5p*	10763.4	5651.0
*hsa-mir-194-5p*	72.4	8209.3
*hsa-mir-205-5p*	291365	11835
*hsa-mir-215*	1152.4	69250
*hsa-mir-145-5p*	16925.6	1.0681e + 05
*hsa-let-7a-5p*	27926.4	20798
*hsa-mir-192-5p*	4710.6	2.4061e + 05

### Next generation sequencing

RNA (<70 nucleotides) was subjected to NGS as previously described [6] and read sequences were aligned onto version (v18) of miRBase, a repository of up-to-date miRNA information of many species including human. Alignment was performed using the bowtie short-read aligner software (version 0.12.7). NGS read counts for a specific miRNA were expressed as number of counts for that miRNA/million miRNA reads. After normalized read counts were obtained, a state of the art statistical model for NGS differential expression analysis “R” package called DESeq [7] was used. MicroRNA with p-values <0.05 (adjusted for false discovery rate of 5%) were considered differentially expressed.

### Quantitative real-time polymerase chain reaction

Quantitative real-time polymerase chain reaction (qRT-PCR) was performed as described previously [6] using 50 ηg RNA in custom designed low density array plates from Applied Biosystems. Each sample was run in triplicate and the mean of this technical replicate was used in subsequent calculations. The threshold cycles (Cq) were set to be in the doubling phase of the PCR amplification runs. The Cq values for the target amplicon were normalized by subtracting the Cq value of RNU6B to create a delta Cq. This delta Cq was used to determine the relative fold differences using the delta-delta Cq method.

### Statistical analysis

Pearson’s correlation coefficients were calculated for the log_2_ transformed, normalized copy numbers by NGS and Cq and delta Cq values by qRT-PCR. Fold changes on NGS and qRT-PCR were compared. We also calculated the accuracy, precision and recall of NGS for the differentially expressed miRNA considering qRT-PCR as the gold standard. A miRNA was labeled as differentially expressed by qRT-PCR in two different ways for purpose of the analysis—either log_2_ fold change > 2 or a p-value <0.05. True positives were defined as differentially expressed miRNA on NGS as well as qRT-PCR with the same direction of fold change. False positives were defined as differentially expressed miRNA on NGS but not by qRT-PCR or if the direction of fold change was opposite between NGS and qRT-PCR. Descriptive statistics were employed to evaluate the NGS dataset for a threshold copy number for reliable qRT-PCR detection. A p value of <0.05 was considered significant.

## Results

The average NGS read counts (reads per million) for all miRNA in BE samples were 1060 per sample, median 3.3, 25^th^-75^th^ percentile 0.74-26.8 (range 0.59-298,713.3). The average NGS read counts for all miRNA in GERD samples were 1415 per sample, median 3.5, 25^th^-75^th^ percentile 0.87-27.5 (range 0.63-614,409.9). The normalized data were previously deposited at NCBI bioproject repository (accession# PRJNA178304) (http://www.ncbi.nlm.nih.gov/bioproject) [6]. We found that the overall correlation coefficients between NGS reads and Cq cycles for BE and GERD patients in the initial cohort of 11 patients were -0.37 (-0.33 to -0.52) and -0.33 (-0.31 to -0.47) respectively, both p < 0.05. We subcategorized miRNA expression based on NGS read counts and compared PCR results across these categories (Table 2). The Cq values were inversely proportional to the NGS read count. For reads > 1000, Cq values increased by ~ two cycles for every 10-fold increase in NGS reads. Since Cq cycles are logarithmic, a change of two cycles indicates a fourfold change in abundance of the particular miRNA (Table 2). We also categorized miRNAs based on their Cq values and found that the NGS read counts progressively decreased with increasing Cq values (Table 3a). Of note, if the Cq values were higher than 35, the average NGS reads were much lower (Table 3b). Thus, a low-abundance transcript on PCR is likely to have low abundance by NGS. However, vice versa is not true. Cq cycles were still in the range of 28-29 for low NGS reads of 1-100 (Table 2). Whether these miRNA of low abundance by NGS are of biological significance needs to be examined.

**Table 2:** NGS read counts and distribution of Cq values

**NGS reads**	**Average Cq values**	**Average delta Cq values**
0-10	29.7	8.6
11-100	28.5	9.1
101-1000	29.1	7.7
1001-10000	27.2	6.6
10001-100000	25.3	3.2
>100000	21.1	1.4

NGS, next generation sequencing, NGS reads for a specific miRNA refers to the counts/million miRNA reads.

Cq, threshold value on qRT-PCR.

deltaCq, Cq_(miRNA)_-Cq_(RNU6B)._

**Table 3:** Distribution of NGS reads based on Cq and delta Cq values

**Table 3a**		**Table 3b**	
***Cq values***	***Average NGS reads***	***delta Cq values***	***Average NGS reads***
<20	54996	<0	50428
20-24	75764	0-4	30414
25-29	2621	5-9	1485
30-34	2466	10-14	955
35-39	383	15-19	435

NGS, next generation sequencing.

Cq, threshold value on qRT-PCR.

delta Cq, Cq_(miRNA)_-Cq_(RNU6B)_.

**Figure 1:**
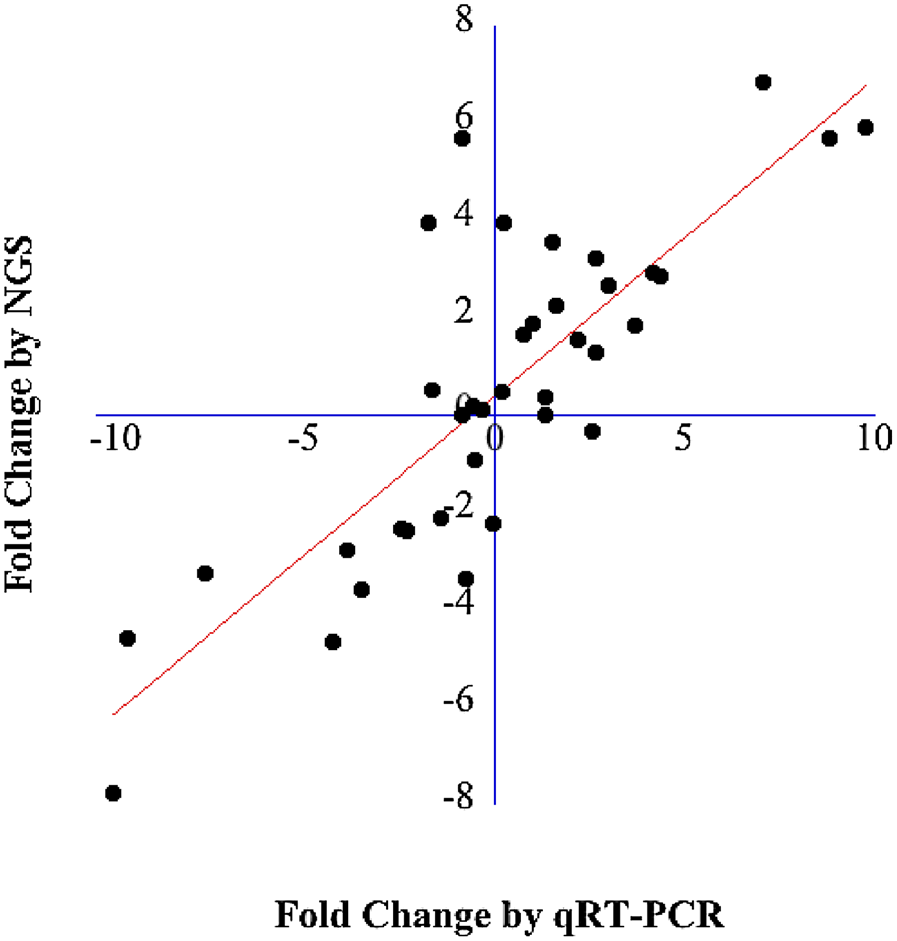
**Graph depicts the correlation between fold changes for the individual miRNA expression values by next generation sequencing (NGS) and qRT-PCR.** The fold changes by NGS were log_2_ transformed. The line highlights the degree of fit indicating a high correlation.

The primary purpose of a high-throughput technology is to detect molecular changes across groups. Presumably the differentially expressed molecular factors are the ones likely to be associated with the observed phenotype. We validated the initial NGS results in an independent validation cohort of 47 patients. Overall, the validation rate by qRT-PCR of differentially expressed miRNA by NGS was 73%. We compared fold changes between BE/GERD by NGS to the fold changes predicted by qRT-PCR and found the correlation to be high, 0.86 (0.68-0.9, p = 2.45E-11) (Figure 1). We did not find any difference in the correlation of fold changes when different thresholds of miRNA expression by NGS were compared. Correlation coefficients were 0.84 (0.57-0.94) vs. 0.80 (0.56-0.91) for miRNA with NGS reads ≥ 1000 versus <1000, 0.82 (0.58-0.93) vs. 0.81 (0.58-0.92) for reads ≥ 500 versus <500 and 0.80 (0.57-0.91) versus 0.89 (0.76-0.98) for reads ≥100 versus <100.

We also calculated the performance characteristics of NGS compared to qRT-PCR. We used two different criteria, first, we used a p value of <0.05 on PCR to define differential expression. Based on the p-value criteria, NGS had an accuracy of 0.71, precision of 0.87 and recall of 0.74 with an f-measure of 0.80. Second, we used a commonly applied criterion of 2-fold change to define differential expression. Based on the fold change criteria, NGS had an accuracy of 0.75, precision of 0.88 and recall of 0.79 with an f-measure of 0.83.

## Discussion

To summarize, we made two main observations—first, although there is a significant correlation between the NGS read counts and PCR Cq values, NGS is only modestly accurate at absolute quantification and second, there was a high degree of correlation between NGS and PCR in fold changes for differentially expressed miRNAs across the GERD and BE groups. This correlation was similar for low-abundance versus high-abundance transcripts by NGS. These findings are significant for investigators focused on making miRNA discoveries driving a disease state as NGS datasets are generally limited because of cost restraints. The differences in accuracy for absolute versus relative quantification can be explained on the basis of bias introduced by the library preparation method [8]. The library preparation method may preferentially amplify some miRNAs but this bias is miRNA-specific and systematic across biologic states thus allowing for differential expression to be robust. Arguably, the differential expression metric is the most biologically relevant.

qRT-PCR and hybridization-based arrays are other methods for high-throughput miRNA detection. Several studies have compared NGS and qRT-PCR for miRNA expression [1-4]. However, the published studies do not provide enough quantitative details with regards to performance of low- versus high-abundance transcripts by NGS. Others are limited by semi-quantitative analysis and validation biased towards miRNA transcripts found to be differentially expressed by NGS [3]. Validation of only those miRNAs differentially expressed by NGS may overestimate its performance. Considering hybridization-based microarrays, studies suggest platform dependent performance for microarrays [1,9].

An important parameter for a high-throughput method is its validation rate. Our overall validation rate for NGS was 73%, significantly higher than the validation rates of 30-40% reported for microarray based methods [10,11]. A potential microarray limitation is its reduced ability to detect differential expression at low expression levels of the miRNA [9]. NGS fold changes did not depend on the expression level in the current dataset. Thus, NGS may have an advantage over microarray for evaluation of low abundance transcripts. With decreasing costs, potential for identification of novel transcripts and further standardization of NGS methods, NGS is likely to replace miRNA microarrays as the technique of choice for high-throughput analysis of miRNA expression.

Our study has some limitations. We studied SOLiD but not the more prevalent Illumina sequencing platform. NGS technology is costly. Also, NGS requires considerable RNA input that makes it difficult to test multiple platforms simultaneously. qRT-PCR may not be the perfect gold standard compared to techniques such as northern blotting and cloning but it is commonly used to validate NGS results prior to embarking on the functional studies. Our study argues that the step of PCR validation may not be necessary if the primary goal is to identify miRNAs that change between control and disease states. A “spike-in” test using synthetic miRNAs could have been useful but would have controlled for technical but not biological variance. As discussed earlier, the library preparation during NGS may be biased towards specific miRNAs but this bias affects specific miRNAs and not specific samples. Inclusion of a few artificial spike-in tests would have not controlled for the miRNA specific effect of the library preparation method and would not have changed the overall conclusions.

## Conclusions

NGS has modest correlation with quantitative PCR for absolute quantification but high correlation for differential expression across the comparison groups. NGS has a high validation rate for the differentially expressed miRNAs. Thus, NGS is ideally suited for biologic studies to further understand the role of miRNA in premalignant gastrointestinal neoplasia.

## Availability of supporting data

The normalized next generation sequencing data were previously deposited at NCBI bioproject repository (accession# PRJNA178304) (http://www.ncbi.nlm.nih.gov/bioproject) [6].

## Abbreviations

NGS: Next generation sequencing; miRNA: MicroRNA (we avoided abbreviating miRNA at the beginning of a sentence to improve readability and appearance); BE: Barrett’s esophagus; GERD: Gastroesophageal Reflux Disease; qRT-PCR: Quantitative real-time polymerase chain reaction.

## Competing interests

The authors declare that they have no competing interests.

## Authors’ contributions

IHL performed the sequence alignment, performed the statistical analysis and drafted the manuscript. XH performed the RNA isolation and PCR experiments. SCM reviewed all the biopsies to confirm the diagnosis of Barrett’s esophagus and provided important feedback. MS critically reviewed the manuscript and made suggestions to the presentation of the data. AR and PS contributed to the repository and provided important feedback to improve the composition of the manuscript. LKC participated in the design of the study, supervised the PCR experiments, and helped to draft the manuscript. AB conceived of the study, and participated in its design and coordination and drafted the manuscript. All authors read and approved the final manuscript.

## References

[B1] Git A Dvinge H Salmon-Divon M Osborne M Kutter C Hadfield J Bertone P Caldas C Systematic comparison of microarray profiling, real-time PCR, and next-generation sequencing technologies for measuring differential microRNA expression RNA 2010 16 5 991 1006 10.1261/rna.1947110 20360395 PMC2856892

[B2] Kolbert CP Feddersen RM Rakhshan F Grill DE Simon G Middha S Jang JS Simon V Schultz DA Zschunke M Kolbert CP Feddersen RM Rakhshan F Grill DE Simon G Middha S Jang JS Simon V Schultz DA Zschunke M Lingle W Carr JM Thompson EA Oberg AL Eckloff BW Wieben ED Li P Yang P Jen J Multi-platform analysis of microRNA expression measurements in RNA from fresh frozen and FFPE tissues PLoS One 2013 8 1 e52517 10.1371/journal.pone.0052517 23382819 PMC3561362

[B3] Kozubek J Ma Z Fleming E Duggan T Wu R Shin DG Dadras SS In-depth characterization of microRNA transcriptome in melanoma PLoS One 2013 8 9 e72699 10.1371/journal.pone.0072699 24023765 PMC3762816

[B4] Schulte JH Marschall T Martin M Rosenstiel P Mestdagh P Schlierf S Thor T Vandesompele J Eggert A Schreiber S Schulte JH Marschall T Martin M Rosenstiel P Mestdagh P Schlierf S Thor T Vandesompele J Eggert A Schreiber S Rahmann S Schramm A Deep sequencing reveals differential expression of microRNAs in favorable versus unfavorable neuroblastoma Nucleic Acids Res 2010 38 17 5919 5928 10.1093/nar/gkq342 20466808 PMC2943620

[B5] Sharma P Clinical practice: Barrett's esophagus N Engl J Med 2009 361 26 2548 2556 10.1056/NEJMcp0902173 20032324

[B6] Bansal A Lee IH Hong X Mathur SC Tawfik O Rastogi A Buttar N Visvanathan M Sharma P Christenson LK Discovery and validation of Barrett’s Esophagus MicroRNA transcriptome by next generation sequencing PLoS One 2013 8 1 e54240 10.1371/journal.pone.0054240 23372692 PMC3553128

[B7] Anders S Huber W Differential expression analysis for sequence count data Genome Biol 2010 11 10 R106 10.1186/gb-2010-11-10-r106 20979621 PMC3218662

[B8] Linsen SE de Wit E Janssens G Heater S Chapman L Parkin RK Fritz B Wyman SK de Bruijn E Voest EE Linsen SE de Wit E Janssens G Heater S Chapman L Parkin RK Fritz B Wyman SK de Bruijn E Voest EE Kuersten S Tewari M Cuppen E Limitations and possibilities of small RNA digital gene expression profiling Nat Methods 2009 6 7 474 476 10.1038/nmeth0709-474 19564845

[B9] Wang Y Barbacioru C Hyland F Xiao W Hunkapiller KL Blake J Chan F Gonzalez C Zhang L Samaha RR Large scale real-time PCR validation on gene expression measurements from two commercial long-oligonucleotide microarrays BMC Genomics 2006 7 59 10.1186/1471-2164-7-59 16551369 PMC1435885

[B10] Bansal A Lee IH Hong X Anand V Mathur SC Gaddam S Rastogi A Wani SB Gupta N Visvanathan M Bansal A Lee IH Hong X Anand V Mathur SC Gaddam S Rastogi A Wani SB Gupta N Visvanathan M Sharma P Christenson LK Feasibility of MicroRNAs as biomarkers for Barrett’s Esophagus progression: a pilot cross-sectional, phase 2 biomarker study Am J Gastroenterol 2011 106 6 1055 1063 10.1038/ajg.2011.37 21407181

[B11] Ozsolak F Milos PM RNA sequencing: advances, challenges and opportunities Nat Rev Genet 2011 12 2 87 98 10.1038/nrg2934 21191423 PMC3031867

